# Promoting smoking abstinence in smokers willing to quit smoking through virtual reality-approach bias retraining: a study protocol for a randomized controlled trial

**DOI:** 10.1186/s13063-020-4098-5

**Published:** 2020-02-26

**Authors:** Alla Machulska, Tanja Joan Eiler, Armin Grünewald, Rainer Brück, Katharina Jahn, Björn Niehaves, Heiko Ullrich, Tim Klucken

**Affiliations:** 1 Department of Clinical Psychology, University of Siegen, Adolf-Reichwein-Str. 2a, 57068 Siegen, Germany; 2 Department of Medical Informatics und Microsystems Engineering, University of Siegen, Siegen, Germany; 3 Life Science Faculty, University of Siegen, Siegen, Germany; 4 Department of Business Informatics, University of Siegen, Siegen, Germany; 5 Department of Psychiatry, Psychotherapy and Psychosomatics, Kreisklinikum Siegen GmbH, Siegen, Germany

**Keywords:** Approach bias, Approach bias modification, Virtual reality, Cigarette smoking, Nicotine addiction, Randomized control trial

## Abstract

**Background:**

Automatic processes to approach smoking-related cues have been repeatedly linked to smoking status, intensity of smoking, and cigarette craving. Moreover, recent findings suggest that targeting those tendencies directly by means of approach bias modification (ABM) has merit in changing maladaptive approach tendencies for drug cues and reducing drug consumption. However, training effects tend to be small. Embedding the training into virtual reality (VR) technology could be a promising way to improve training efficacy. The present protocol describes a randomized controlled trial that aims to assess the efficacy of a newly developed VR-ABM as a means of reducing smoking-related approach biases or nicotine consumption in smokers seeking abstinence.

**Methods:**

One hundred daily smokers who are motivated to quit smoking will be recruited into the randomized controlled trial. All participants will attend a brief smoking cessation intervention (TAU) and will be randomly assigned either to the experimental (VR-avoidance training) or the placebo-control group (VR-placebo training). During the VR-avoidance training, participants are implicitly instructed to make an avoidance movement in response to smoking-related objects (e.g., cigarettes) and an approach movement in response to alternative objects (e.g., healthy food). During the VR-placebo training, no such contingency between arm movement and item content exists. Trainings are administered in six sessions within two weeks. Training effects on automatic approach tendencies and smoking behavior are measured immediately after training and at a 7-week follow-up.

**Discussion:**

Embedding the training into virtual reality (VR) technology could be a promising new way to improve ecological validity, realism, and immersion and thereby increase ABM training effects. The results of this study can inform future research in the optimization and advancement of treatment for addiction.

**Trial registration:**

Registered with Current Controlled Trials: study ID ISRCTN16006023. Registered on 28 March 2019.

## Background

Tobacco smoking remains the most preventable cause of premature death and morbidity worldwide. While most smokers are aware of the detrimental effects related to smoking, and most smokers desire to quit smoking, common behavioral interventions for smoking are of limited efficacy. Even following effective treatment, fewer than 25% of smokers manage to remain abstinent for at least 6 months [1–3]. Thus, an urgent need exists for the development of more effective treatment options and the improvement of efficacy of existing smoking cessation interventions.

One potential reason for these discouraging outcomes might be that addictive behavior is not only driven by explicit, rational, or reflective processes that are usually targeted, by traditional treatments but also by implicit, hard to control, automatic processes [4, 5]. In line with this notion, dual process models of addiction [6, 7] propose that both limited reflective processes *and* strengthened impulsive processes are the key determinants of addiction development, maintenance, and treatment. The latter processes are supposed to be implicated in the formation of so-called information-processing or *cognitive* biases. That is, as addiction progresses, information processing is biased in favor of drug-related cues, making drug taking more likely. The approach bias for smoking can be defined as the automatic tendency to approach smoking-related cues and is of special interest because of its strong connection with cigarette craving and actual smoking behavior [8, 9].

Most recently, the Approach-Avoidance Task (AAT)—originally designed to assess automatic avoidance tendencies in spider-anxious individuals [10]—has proven useful in both measuring and modifying approach biases for appetitive behaviors, including smoking [8, 9]. During the task, various images appear on a computer screen. Participants are instructed to ignore image content and to respond to a content-irrelevant feature such as image format or orientation by either pulling or pushing a joystick attached to the computer. Upon a pull movement, the picture increases in size, whereas upon a push movement, the picture shrinks. This zooming feature disambiguates the task and creates a visual sense of approach and avoidance. An approach bias is inferred from faster pulling than pushing of a picture of interest. Hence, the AAT can be viewed as a measure of performance deficits due to the incongruence between the instructed movement and the automatically triggered response. Using the AAT, an approach bias for cigarette cues was found in smokers but not in ex-smokers or those who have never smoked [9]. A study conducted by Watson and colleagues [11] showed that acute exposure to nicotine (i.e., smoking a cigarette) led to an increased nicotine-related approach bias. Finally, a study by our own working group [8] showed that smokers display an automatic approach bias for smoking cues but not for natural rewards (such as highly palatable food).

Throughout the last decade, a large body of research has shown that experimental tasks originally developed to measure cognitive biases also have merit in modifying such biases, sometimes leading to improved health behavior [4]. For instance, the AAT has been adapted to a training variant by changing the contingency between picture content and arm movements. In the training version of the task, smoking-related pictures are consequently presented in the push-away format, while alternative pictures are presented in the pull-closer format. Recently, we applied four sessions of AAT-training as an add-on to a brief smoking cessation intervention to a sample of inpatient psychiatric smokers [12]. Compared to sham training, the AAT-training led to a larger reduction of nicotine consumption at a 3-month follow-up. In a related study, smokers seeking abstinence were assigned to four sessions of AAT-training or sham training and were instructed to make a self-guided quit attempt upon completion of the final training session [13]. The reduction in approach bias emerged as being positively related to the number of days abstinent following the quit attempt. Hence, upcoming evidence suggests that approach bias retraining by means of the AAT might be a useful intervention to reduce smoking or promote abstinence. Similarly, several reviews and meta-analyses support the above-mentioned results and give rise to the premise that approach bias retraining has merit in changing addictive behaviors [4, 5]. However, the effects tend to be small or sometimes even mixed [14]. Thus, improving training efficacy constitutes a key challenge for cognitive bias modification (CBM) programs.

Although the exact training mechanisms remain uncertain, cognitive bias trainings apparently must be administered multiple times to generalize to real-world behavior and to produce reliable and stable changes over time. This requires participants to be willing to maintain adherence to a daily or weekly training prescription. However, adherence to the trainings might be limited, as trainings are often perceived as monotonous or boring. Confirming the above-mentioned concerns, preliminary evidence suggests that high dropout rates can be challenging for CBM studies [12, 15, 16]. Another reason for rather small training effects concerns the generalization of training to actual everyday behavior. It has been argued that the interaction with simple two-dimensional images reduces the realism of the training and thereby narrows the ecological validity of the task [17]. Virtual reality (VR) simulates a natural environment by presenting an interactive three-dimensional space that mirrors everyday-like arm or posture movements [18]. The realism and immersion created by VR can reasonably be assumed as possibly contributing to the ecological validity and enhancing the engagement, enjoyment, and—most importantly—training adherence [19]. Therefore, this study introduces a novel VR approach bias training (VR-AAT) for smoking as an add-on to brief behavioral counseling for smoking cessation. Our objective is to investigate whether the newly developed VR-AAT is capable of modifying existing approach biases for smoking cues and/or changing smoking behavior by promoting abstinence or reduction of nicotine consumption. The study utilizes a randomized placebo-control design and multi-session VR training during a 2-week training period. We expect that the VR-AAT would reduce smoking-related approach biases as measured with the assessment version of the AAT [8], reduce the number of daily smoked cigarettes, and increase abstinence rates over and above the placebo-control condition.

## Methods/Design

### Trial design

The present study is a randomized control trial comparing self-report, behavioral, and biochemical outcomes of the VR-AAT training to an active placebo control condition (Additional file 1). Smokers are randomly assigned either to the experimental (brief smoking cessation intervention, hereafter referred to TAU + VR-avoidance training) or to the sham-control condition (TAU + VR-placebo training). This study employs a 2 (condition: avoidance training vs. placebo training) × 3 (time: pretest/post-test: 3 weeks after baseline/follow-up: 7 weeks after baseline) mixed design. Because we expect the experimental condition to be more effective than the sham-training control condition, this protocol describes a superiority trial design. The training intervention occurs over the course of 2 weeks in which smokers complete six training sessions in total. Measures of cognitive biases (approach, attention, and association bias), self-report measures of smoking behavior and biochemically verified smoking intensity are applied at the pretest, post-intervention, and 7-week follow-up.

A priori power analyses were conducted using G*Power 3.1 (open-source software) [20]. Previous research on the efficacy of CBM trainings found a small to moderate effect size [12, 21]. We conducted a power analysis for a 2 × 3 mixed design ANOVA to detect a small-to-moderate effect (Cohen’s d = .30). Our results indicated that a total sample size of 74 is required to achieve 80% power at an alpha level of 0.05 and an assumed correlation of r = .50 between the repeated measures. Because we expect some attrition due to our multisession design, we decided to include 100 participants, which is 50 per condition.

### Participants

**Fig. 1:**
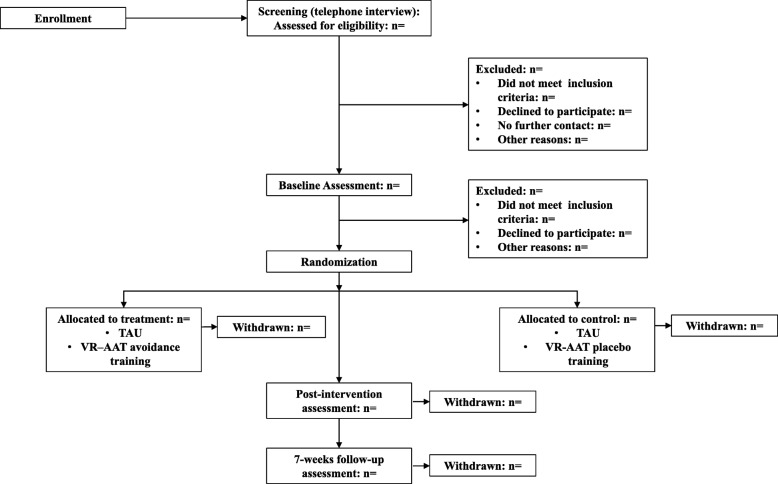
CONSORT flow diagram

Current smokers (*n* = 100) will be recruited at the University of Siegen (Germany) and from the general population. Figure 1 provides a CONSORT diagram of the participant recruitment. Participants will be recruited through flyer advertisements, radio broadcasts, television reports, newspaper reports, and web advertisement. Interested participants will receive information regarding the study via e-mail and will be invited to take part in a telephone interview to assess eligibility criteria. Smokers will be included if they smoked more than six cigarettes per day during the last 6 months. Exclusion criteria are current alcohol or drug misuse or dependency, psychiatric illness, insufficient German language skills, or uncorrected visual or auditory impairment. Participants enrolled in the study complete nine sessions in total, which are composed of three laboratory assessment sessions (pretest, post-test, and 7-week follow-up) and six VR-training sessions (carried out between the pretest and post-test). Full written informed consent will be obtained from each participant at study entry.

### Ethics statement

The study protocol (version 1: 05/2019) was approved by the local Ethics Committee of the University of Siegen (reference number: ER_18_2018) and was conducted in accordance with the Declaration of Helsinki and Good Clinical Practice guidelines. During the course of the study, ethical, legal, and social aspects (ELSA) will be anticipated and addressed. Each participant will provide a written informed consent to the experimental procedure prior to the inclusion in our study. Participation will be entirely voluntary and participants will have the right to withdraw their consent for participation at any time.

### Randomization and blinding

Participants will be randomly assigned to the experimental or placebo control group, according to an externally constructed randomization plan. The ratio will be 1:1. A computer-generated randomization schedule will be employed by means of a computerized random number generator using IBM SPSS Statistics 24. Permuted block randomization will be used to ensure that study groups of approximately the same size are generated. Block size will be set to 50 participants. No other stratification will be used. Although participants will be blind to the experimental conditions, blinding of the assessors is not possible because of the course of the study (single-blind trial). In addition, a training evaluation questionnaire will be employed at post-test and follow-up, which will serve as a manipulation check in order to examine whether single-blinding was possible. To avoid bias in the outcome assessment, research assistants concerned with data collection and/or preparation will be blind to the allocation of the participants.

### Intervention

Prior to the training, participants take part in a brief psychoeducational workshop containing information on nicotine addiction, the short- and long-term effects associated with cigarette smoking, and behavioral approaches to smoking cessation (approximately 60 min). Afterward, smokers receive a self-help book (“The Easy Way to Stop Smoking” by Allen Carr) to aid smoking cessation and will be instructed to record their daily intake of cigarettes by using a mobile phone application specifically developed for this study (Cigarette Tracking List, SenseAble UG). According to the dual-process models of addiction, these behavioral approaches should target reflective processes associated with smoking, resemble traditional smoking cessation interventions, and therefore constitute our TAU intervention. To address more automatic, impulsive processes, participants receive either the VR-avoidance training or the VR-placebo training according to the predefined randomization plan.

### VR setup and experimental manipulation

To modify smoking-related approach biases, we apply a novel VR setup, which is based on the AAT-training previously used by our research group [12]. Participants perform trainings in a standing position and are equipped with HTC Vive HMD, which has a resolution of 2160 × 1200 pixels and a refresh rate of 90 Hz (HTC Corporation, ViveTM). The Leap Motion infrared sensor (Leap Motion, Inc., San Francisco, CA) is used to transfer the participants’ own hand movements into the VR, thereby enhancing body ownership and immersion. The training was implemented using the Unity3D engine; therefore, the C# language was used for programming the scripts. Smoking and control items were obtained from the Unity3D asset store and were modified in terms of color and size.

**Fig. 2:**
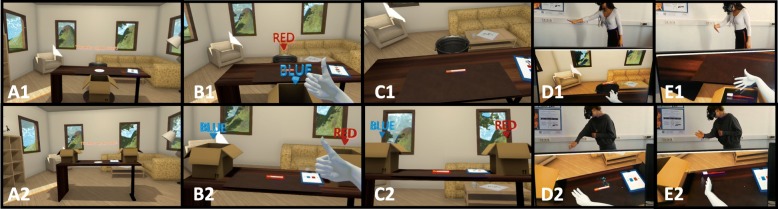
Virtual reality setting. (A) VR environment. The upper line of the Fig. 1 shows the VR-AAT avoidance condition, the bottom line (2) shows the VR-AAT placebo condition. (B) Thumb-up gesture to start the training. (C) Participants’ view of a smoking-related item (here: cigarette). (D) Participant equipped with the HTC Vive head-mounted display and interacting with a smoking-related VR item: (1) pushing a cigarette in the VR-AAT avoidance condition vs. (2) sorting a cigarette to the right in the VR-AAT placebo condition. (E) Participant interacting with a smoking-unrelated VR item: (1) grasping a toothbrush in the VR-avoidance condition vs. (2) sorting a toothbrush to the left in the VR-placebo condition.

The VR scenario places participants inside a virtual office space in which they are instructed to interact with virtual smoking-related objects and nonsmoking control objects using their dominant hand (*see* Fig. 2). Each training begins with the participant making a thumbs-up gesture. During the trial, 10 different smoking (i.e., cigarette, cigar, cigarette lighter, cigarette pack, or ashtray) and 10 shape- and color-matched control objects (pen, toothbrush, mug, drinking bottle, book, tennis ball, apple, or avocado) appear consecutively in the middle of a virtual desk. The items are bordered in either red or blue. Participants are asked to ignore item content and to respond to border color by using their own hand.

In the VR-avoidance training, smokers are instructed to respond to red-bordered objects by making a warding arm movement and throwing them as quickly as possible into a trashcan placed behind the desk. In addition, they should respond to blue-bordered objects by making a grasping movement and placing them as quickly as possible into a box in front of the desk. Importantly, all smoking-related objects are bordered in red and must be avoided, whereas all smoking-unrelated objects are bordered in blue and must be grasped. Thus, consistent with other CBM studies, an indirect instruction was employed.

The VR-placebo training is similar to the VR-avoidance training in that it requires participants to ignore item content and to react to border color. However, this task abstains from using any approach (i.e., grasping) or avoidance (i.e., warding) movements. That is, participants are asked to respond as quickly as possible to the objects appearing on the desk by placing blue-bordered objects into a box positioned on the left-hand side of the desk and red-bordered objects into a box on the right-hand side of the desk. In addition, no contingency between item content and box position existed.

To ensure training fidelity, a cover story is used in both conditions in which participants receive a credible explanation for the training being arranged as it is. That is, participants in the VR-avoidance training will be told that the training constitutes an avoidance training, while participants in the VR-placebo training are told that the training constitutes a cognitive control training. To ensure that both cover stories are plausible to the participants, we will apply a training evaluation questionnaire of the VR training program in the post-test and follow-up assessments.

Participants must execute the correct movement to make the item disappear. Each item is shown six times, resulting in 120 trials per training. Training sessions take approximately 15 min to complete.

### Material and measures

#### Cognitive bias assessment

The approach, attentional, and association biases for smoking will be measured at pretest, post-test, and follow-up to investigate whether the VR-training is capable of changing existing cognitive biases associated with smoking.

#### Approach bias assessment

Automatic approach biases for smoking-related stimuli will be measured by means of both the standard version of the joystick-AAT (see [8]) and the VR-based AAT. During the standard AAT, 25 smoking-related and 25 positive images derived from Baird et al. [13] appear on a computer screen. Images are tilted 3° to the left or 3° to the right. A joystick (Logitech Extreme 3D) is connected to the computer, and participants are told to use the joystick to push images rotated to the right and to pull images rotated to the left. As a result, images shrink or grow in size depending on the arm movement. For the purpose of bias assessment, smoking and control pictures have to be pulled and pushed equally often. Each picture is shown once in push-away format und once in pull-closer format, resulting in 100 assessment trials. Approach biases derived from the standard AAT are computed for each of the laboratory sessions (pretest, post-test, 7-week follow-up).

The assessment version of the VR-AAT is similar to the VR-avoidance training, with the exception that the 10 smoking-related and 10 smoking-unrelated objects must be grasped and warded off equally often, thereby enabling bias assessment. Each item appears three times in a grasping format and three times in a warding off format, resulting in 120 assessment trials.

An approach bias is inferred from faster pulling/grasping than pushing/warding an item. That is, an approach bias score is calculated by subtracting median reaction times (RT) for pulling an item from median RTs for pushing the exact same item. The reaction time is defined as the time in milliseconds required by a participant to execute the correct full arm movement. Accordingly, a positive value indicates an approach tendency toward a picture category, whereas a negative value indicates an avoidance tendency.

#### Attentional bias assessment

Automatic attention biases for smoking cues will be measured by a visual dot probe task adapted from Miller and Fillmore [22]. The task will be operated using Inquisit Lab software and will be performed on a personal computer. Each trial starts with a 500-ms fixation cross in the center of the computer screen. Afterwards, two 13 × 18 cm images (a smoking-related and a control picture) appear side-by-side and 3 cm apart on the left and right side of the computer screen. Image position is randomly chosen to be either left or right of the location of the fixation cross. After 1000 ms, the two pictures disappear, and a probe stimulus (here: “X”) appears in the location of one of the pictures. A response pad (Cedrus Response Pad RB844) is attached to the computer, and participants are instructed to press a yellow key if the probe is left and to press a green key if the probe is right.

The task stimulus consists of 10 smoking-related images that were matched with 10 neutral tooth-cleaning control images. This image set was provided by Stippekohl and colleagues [23] and has been previously used in recent studies conducted by members of our research group [8, 12, 24]. This pictures bring the advantage of being carefully matched in terms of colors and shape (see [23]). In addition, all images contain a simple smoking or tooth-cleaning behavior without any distractors. This is crucial since evidence hints to the fact that complex addiction-related scenes might be less effective at capturing participants’ attention and could therefore result in less attentional bias when used in visual probe tasks [22]. Each image pair is presented four times, resulting in 40 test trials. In addition, 40 filler trials are included that consisted of 10 pairs of neutral images. This approach is used to reduce possible habituation to smoking-related stimuli that might occur otherwise [25]. Test and filler trials are presented randomly, resulting in 80 trials in total. Filler trials are not included in the final data analysis.

An attentional bias is inferred from faster reaction times to probes that replace a smoking-related image relative to those that replace a tooth-cleaning control image. For the calculation of an attention bias score, RTs for probes replacing the smoking pictures were subtracted from RTs for probes replacing the tooth-cleaning pictures. Thus, a positive value indicates an attention bias toward smoking-related pictures, whereas a negative value indicates an attention bias in favor of tooth-cleaning pictures.

#### Association bias assessment

Implicit positive or negative associations for smoking will be assessed by an implicit association task (IAT [26];). The task is adapted from Kahler and colleagues [27]. Participants are instructed to categorize positive and negative attributes (e.g., “trustful” vs. “hurtful”) and target items (e.g., images of smoking-related objects or furniture, i.e., a cigarette vs. a chair) into predetermined categories via response pad button presses (Cedrus Response Pad RB844).

Following Greenwald et al. [28], the IAT is organized into seven blocks: (a) a 24-trial target discrimination block (e.g., press yellow for “smoking” vs. press green for “furniture”); (b) a 24-trial attribute discrimination block (e.g., yellow for “I feel positive” vs. green for “I feel negative”); (c) a 24-trial practice combined block (e.g., yellow for “smoking” OR “I feel positive” vs. green for “furniture” OR “I feel negative”); (d) a 40-trial test combined block (same as practice); (e) a 24-trial target discrimination block, in which the target categories are reversed (e.g., yellow for “furniture” vs. green for “smoking”); (f) a 24-practice combined block with reversed target categories (e.g., yellow for “furniture” OR “I feel positive” vs. green for “smoking” OR “I feel negative”), and (g) a 40-trial test combined block (same as practice). Blocks c, d, f, and g are crucial blocks used in scoring the IAT.

Because we assume that smokers associate smoking with positive feelings, trials in which smoking and positive consequences share a response key are congruent, whereas trials in which smoking and negative consequences share a response key are incongruent.

The IAT is counterbalanced in two ways to prevent methodological confounds. First, the placements of the target and attribute stimuli labels are counterbalanced (yellow or green button). Second, two IAT orders are used: one with the congruent combination block first and one with the incongruent combination block first.

The IAT bias score is calculated by subtracting RTs for incongruent blocks (i.e., smoking + negative; furniture + positive) from RTs for congruent blocks (i.e., smoking + positive; furniture + negative). Larger IAT scores suggest stronger, implicit, positive, social associations with smoking or stronger, implicit, negative associations with furniture.

### Biochemical verification

Expired carbon monoxide (CO) will be assessed at baseline, post-training, and 7-week follow-up using a Carbon Monoxide Monitor (piCO™ Smokerlyzer®; Bedfont Scientific Ltd).

### Behavioral and self-report measures

To track daily nicotine consumption, participants are asked to self-monitor their smoking behavior via a cigarette-tracking app, which was specifically designed for the purpose of this study. To do so, participants log each smoked cigarette directly after smoking. In addition, participants will be asked to estimate their average daily nicotine consumption at pretest, following each training session, post-test, and 7-week follow-up.

The questionnaire measures will include the Fagerström Test for Nicotine Dependence (FTND) [29] (German version: [30]); the Stages of Change Scale [31] (German version: [32]); the Thoughts About Abstinence Scale [33]; attitudes toward smoking based on Swanson et al. [34]; cigarette craving on a 6-point Likert-scale, ranging from 0 (“not at all”) to 5 (“very high”); and the Barratt Impulsiveness Scale (BIS) [35]. Finally, participants provide information about various health-related behaviors, including eating habits, sports activities, and attending medical check-ups. At post-test, participants will be also required to evaluate the VR-training and indicate their awareness about training contingency.

### Outcome measures

Primary outcome measures are changes in approach bias, which are measured by the assessment versions of the joystick-AAT and the VR-AAT, reductions in nicotine consumption, and abstinence rates. Secondary outcome measures will be changes in other cognitive biases as measured by a visual dot-probe task and the IAT, motivation to stop smoking, other health-related behavior, expired CO, cigarette craving, and explicit attitudes toward smoking.

### Procedure

**Fig. 3:**
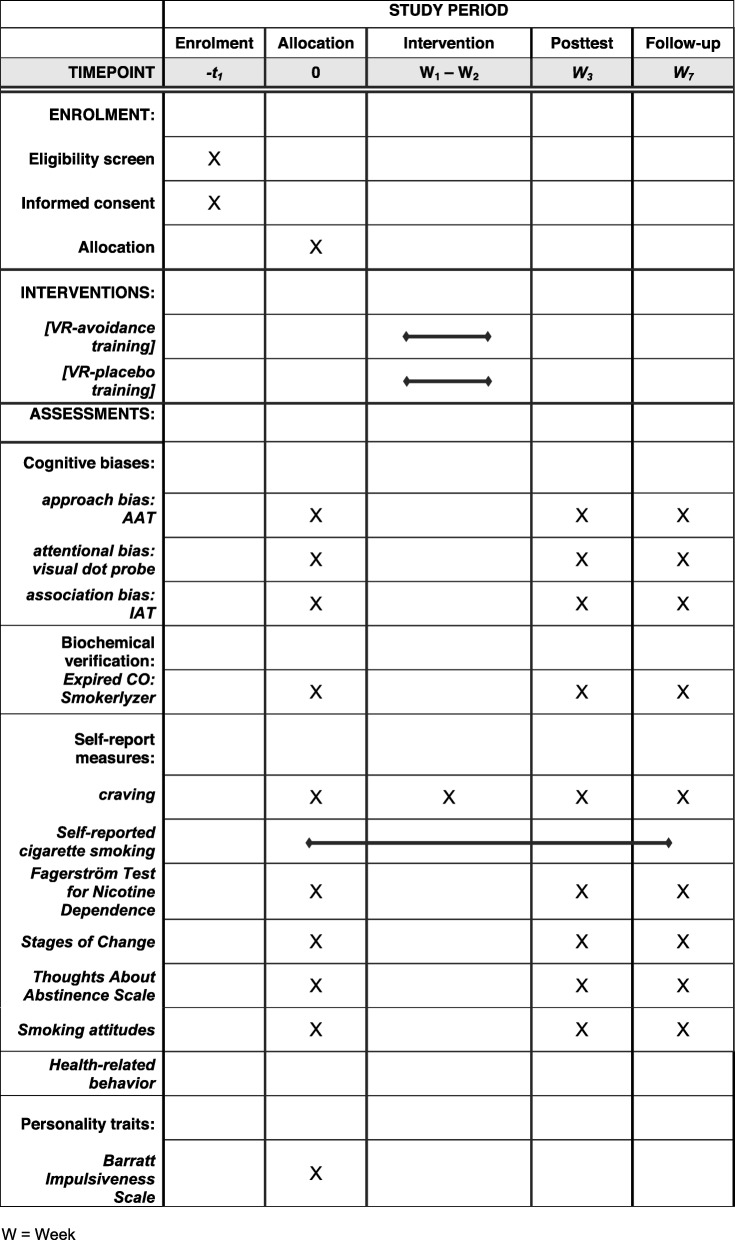
Schedule of enrollment, assessments and interventions

A time schedule of enrollment, assessment visits, and VR-trainings for participants is shown in Fig. 3. During the first laboratory session (baseline; approximately 150 min), participants give informed consent, take part in a short psychoeducational group counselling and receive a self-help book to aid smoking cessation. Individuals are then asked to download the cigarette-tracking app and are instructed to log each smoked cigarette for the duration of their participation in the study. Thereafter, participants complete the cognitive bias assessments, questionnaire measures, and the carbon monoxide breath test. VR-based approach bias assessment takes place prior to the first training session and following the last training session. In total, smokers complete six sessions of VR-training (VR-avoidance or VR-placebo training) within 2 weeks. After completion of the final training session (post-test) and at a 7-week follow-up, participants are once again invited to complete cognitive bias assessment tasks, questionnaire measures, and the CO breath test and are asked to evaluate the training and indicate their awareness about training contingencies. Cigarette craving is measured at pretest, post-test, and follow-up, as well as after each training session, resulting in nine measurement points.

### Data preparation and planned analyses

So that missing data can be filled in, multiple imputation and the modified Intention-To-Treat (ITT) principles [36] will be used. Prior to computation of the cognitive bias scores, error trials will be excluded from further analyses. Median reaction times will be used to minimize the influence of outliers.

To test for changes in cognitive biases, mixed design ANOVAs will be calculated. Due to random assignment to the experimental or to the placebo group, we expect a priori group differences to be small. Possible chance baseline imbalance between treatment arms will be taken into account by means of follow-up simple effects analyses. For approach biases, a 2 (condition: VR-avoidance training vs. VR-placebo training) × 2 (item content: smoking vs. control items) × 3 (time: pretest, post-test, follow-up) mixed design ANOVA will be conducted. For attentional and association biases, two separate 2 (condition) × 3 (time) mixed design ANOVAs will be calculated.

To evaluate the effects of training on self-reported average daily nicotine consumption, a 2 × 3 mixed ANOVA will be carried out in which the training condition is a between-subjects factor and time (pretest, post-test, follow-up) is a within-subjects factor.

Multilevel modeling (MLM) will be used to estimate the growth curve for nicotine consumption as measured with the cigarette-training app and craving ratings over time.

To explore whether the VR-training leads to increased abstinence rates, a Chi-square test will be computed.

To test the effect of training on other smoking-related variables (i.e., CO, FTND, smoking attitude, motivation to quit), a 2 (condition) × 3 (time) repeated measures MANOVA will be conducted. Significant multivariate effects will be followed up with separate ANOVAS on each dependent variable. In case of significant interactions, discriminant factor analyses will be conducted to identify possible associations between each of the dependent variables and group assignment.

## Discussion

The aim of the present study is to explore whether a newly developed VR-training is capable of modifying cognitive biases and/or reducing smoking in terms of nicotine consumption or withdrawal over and above a sham-training control condition. Therefore, we describe a randomized, multisession placebo-control design to explore the efficacy of a VR-based AAT-training in a group of regular smokers willing to quit smoking.

This study is the first to combine approach bias retraining with VR techniques in the realm of addiction treatment. Still, over the last few years, VR approaches have made their ground in more traditional, mostly cognitive-behavioral interventions for psychiatric disorders. Mainly, VR-based techniques have become a promising effective and time-saving alternative for in vivo exposure-based therapy for specific phobias, including dental phobia [37], flight phobia [38], and spider phobia [39]. Moreover, VR has been used for neurocognitive assessments [40] and executive functioning training (for a review, see [41]). Although not used in CBM studies yet, an investigation by Schroeder and colleagues [42] revealed that a VR setting based on the AAT was capable of measuring an approach bias for palatable food in healthy subjects. Results derived from a related study [43] indicate that heavy social drinkers show difficulties in avoiding alcohol-related situations in a virtual environment, whereas no such difficulties were reported for light social drinkers. Hence, while research in this respect is still in its early stages, VR-based techniques hold promise in support of traditional health care.

VR bears several advantages over traditional computer-based techniques, which could offer a useful opportunity to improve the effectiveness of existing CBM programs. For example, VR enables the interaction with 3D-objects and allows for the implementation of complex scenarios that are hardly to provide using traditional PC-based interventions [44]. Thus, certain environments that fit a person’s everyday risk situations can be embedded into the VR-training, thereby narrowing the gap between laboratory trainings and actual real-life behavior. This approach, in turn, has merit in increasing training generalizability and efficacy. In addition, the deployment of more realistic environments can increase the enjoyment, motivation to train, and the feeling of immersion or presence. In turn, high levels of immersion, plausibly, will contribute to adherence and reduce dropout rates [45].

In summary, VR might offer an ecologically valid and promising approach to extend existing cognitive bias trainings, possibly augmenting the efficacy of current CM approaches.

### Trial status

This trial was registered on 28 March 2019 (registration number ISRCTN16006023, protocol version number 1.0). As of November 2019, 108 participants have been enrolled in the study. The first participant was enrolled in 23 March 2019, and completion of recruitment is projected for December 2019. The final results will be published as soon as possible after the analysis is completed.

## Supplementary information

**Additional file 1.** SPIRIT 2013 Checklist: Recommended items to address in a clinical trial protocol and related documents*.

## Data Availability

The datasets used and/or analyzed during the current study are available from the corresponding author on reasonable request.

## Abbreviations

AAT: Approach-avoidance task
ABM: Approach bias modification
ANOVA: Analysis of variance
BIS: Barratt Impulsiveness Scale
CBM: Cognitive bias modification
CO: Carbon monoxide
ELSA: Ethical, legal and social aspects
FTND: Fagerstörm Test for Nicotine Dependence
IAT: Implicit association task
ITT: Intention to treat
MANOVA: Multivariate analysis of variance
MLM: Multilevel modelling
ms: millisecond
RCT: Randomized controlled trial
RT: Reaction time
TAU: Treatment as usual
VR: Virtual reality
